# Balancing Water Resources Development and Environmental Sustainability in Africa: A Review of Recent Research Findings and Applications

**DOI:** 10.1007/s13280-012-0359-1

**Published:** 2012-12-14

**Authors:** Michael E. McClain

**Affiliations:** 1 Department of Water Science and Engineering, UNESCO-IHE Institute for Water Education, Westvest 7, 2611 AX Delft, The Netherlands; 2 Faculty of Civil Engineering and Geosciences, Delft University of Technology, P.O. Box 5048, 2600 GA Delft, The Netherlands

**Keywords:** Sustainable development, Biodiversity conservation, Food security, Hydro-power development, Environmental flows, Climate change

## Abstract

Sustainable development in Africa is dependent on increasing use of the continent’s water resources without significantly degrading ecosystem services that are also fundamental to human wellbeing. This is particularly challenging in Africa because of high spatial and temporal variability in the availability of water resources and limited amounts of total water availability across expansive semi-arid portions of the continent. The challenge is compounded by ambitious targets for increased water use and a rush of international funding to finance development activities. Balancing development with environmental sustainability requires (i) understanding the boundary conditions imposed by the continent’s climate and hydrology today and into the future, (ii) estimating the magnitude and spatial distribution of water use needed to meet development goals, and (iii) understanding the environmental water requirements of affected ecosystems, their current status and potential consequences of increased water use. This article reviews recent advancements in each of these topics and highlights innovative approaches and tools available to support sustainable development. While much remains to be learned, scientific understanding and technology should not be viewed as impediments to sustainable development on the continent.

## Introduction

At the turn of the century, as part of a worldwide visioning process, leaders in Africa formulated Africa Water Vision 2025 (UN-Water 2003). The vision describes the central role of water in Africa’s efforts to address its crippling socio-economic problems and proposes a framework for action (FFA) that sets specific milestones and targets for development of water resources across the continent. Water governance, research, capacity building, and strengthening the financial base are all featured in the FFA, but the most ambitious numeric targets call for doubling the area of irrigated agriculture and increasing fivefold the overall exploitation of water resources for agriculture, hydropower, industry, and domestic needs (Table 1). The importance of sustaining ecosystems and biodiversity is also acknowledged, and the FFA calls for allocation of sufficient water for environmental sustainability across all nations on the continent by 2015, a decade before other targets reach their maximum values. These targets are aligned with numerous national and regional development plans, and they were reaffirmed in a regional paper delivered at the 2009 World Water Forum, where the Africa Development Bank Group called for investments of US $50 billion per year for new water infrastructure to meet targets (WWF5 2009). Actions have been followed up in initiatives such as the African Union’s New Partnership for Africa’s Development (NEPAD 2012), and international donors and investors are responding with several large water-related development programs, sponsored mainly by China, Europe, and the USA (FOCAC 2012; GAFPS 2012; UN-WFP 2012; USAID 2012).

**Table 1:** Milestones and targets for actions under the heading of “Meeting Urgent Water Needs” in Africa Water Vision 2025. Current estimates are that only 7 % of the Africa’s hydropower potential has been developed. Increasing this to 25 % by 2025, while also doubling the area of irrigated agriculture, will increase use of the continent’s water resources by a factor of 5

Actions	Targets	
	2015	2025
Proportion of people without access		
To safe and adequate water supply	Reduce by 75 %	Reduce by 95 %
To safe and adequate sanitation	Reduce by 70 %	Reduce by 95 %
Water for achieving food security		
Water productivity of rain-fed agri. and irrigation	Increase by 30 %	Increase by 60 %
Size of irrigated area	Increase by 50 %	Increase by 100 %
Development of water for agriculture, hydropower, industry, tourism and transportation at national level	10 % of potential	25 % of potential
Conservation and restoration of environment, in biodiversity, and life-supporting ecosystems		
Allocation of sufficient water for environmental sustainability	Implemented in 100 % of countries	Implemented in 100 % of river basins

The need for greater use of water resources is unquestionable, as development across much of Africa over the last half century has lagged far behind that of the rest of the world. Of the 50 least developed countries tracked by the UN’s Human Development Index (UNDP 2011), 34 are in sub-Saharan Africa, including the 15 least developed. More than 300 million sub-Saharan Africans lack access to improved water for domestic use, and approximately 550 million lack access to improved sanitation (WHO/UNICEF 2008). This amounts to 40 and 70 %, respectively, of the total population and the lack of access among rural populations is considerably higher, exacerbating the public health crisis. Agriculture is the main economic activity for a majority of rural Africans, and it is the basis for the food security of rural and urban alike. Agricultural productivity in most of Africa, however, is only a fraction of that on other continents, limiting income from farming and making food security in sub-Saharan Africa among the lowest of any region of the world (FAO 2011). Similarly, sub-Saharan Africa’s power generation capacity is just 68 gigawatts (GW), or 2 % of global capacity. If the capacity of South Africa is omitted, the total of the remaining 47 countries is just 28 GW (Eberhard et al. 2011). Access to electricity is correspondingly low, averaging approximately 20 % of the population, but for rural communities in many countries the figure is closer to 1 % (ADB 2008; Brew-Hammond and Kemausuor 2009). The United Nations estimates that the population in Africa surpassed the one billion mark in 2009–2010, and it projects that population will increase by 33–44 % between 2010 and 2025 (UN-DESA 2010). The needs are therefore growing exponentially, as is the urgency for action.

At the same time, in the absence of many developed services, hundreds of millions of Africans continue to rely heavily on the direct use of ecosystem services to meet basic needs (MEA 2005; Holland et al. 2011). Provisioning ecosystem services such as natural water and food sources, fuel wood, fodder, building materials, and other natural products are essential, and regulating services such as natural soil fertility and water availability strongly influence the level of welfare achievable in a given area. Population growth over the past century has increased pressures on African ecosystems and severe degradation is apparent in many areas (UNDP 2011). As development progresses across the continent, it is necessary to maintain and even restore ecosystem services to support and enhance developed services, to preserve the continent’s extraordinary biodiversity and natural heritage, and to contribute to overall wellbeing. Water is central to this process, and some of the most challenging questions concern how to balance water allocations between extractive, in-stream, and environmental uses.

Over the past few years there has been important progress in research describing the climatic, ecohydrologic, and socioeconomic dimensions of these issues in Africa, and a mix of traditional and innovative approaches are being proposed and tested to enable environmental sustainability going forward. This review considers recent advancements in knowledge of three water-related thematic areas strongly influencing the future of African development. The first is climate and hydrology, especially new understanding of the controls on the spatiotemporal variability of water availability across the continent and projections of future climate change. Second is agriculture and hydropower, including regional and continental projections for growth in these sectors and related demands on African water resources in the coming decades. And third is ecological integrity, with an emphasis on status and threats to the continent’s outstanding ecological features and key ecosystem services. Water for domestic needs, although crucial from a public health and livelihoods perspective, is not considered in this review because abstractions will be small compared to agriculture. Domestic water supply in Africa is more an issue of improving and protecting water sources rather than greatly increasing abstraction. The article concludes by highlighting promising research findings to support balanced development and conservation efforts in the coming decades.

## The Climate Factor and Boundary Conditions for Development

Africa is a dry continent with just 9 %, or 3931 km^3^/year, of world renewable water resources (FAO-Aquastat 2009), compared with 22 % of the world landmass and 15 % of world population. This places constraints on the potential for sustainable development in several regions. Of the continent’s renewable water resources, 72 % is concentrated in central Africa and western regions along the Gulf of Guinea, where 34 % of the population lives. Water availability is generally not a constraint to development in these humid areas, but environmental concerns are high in this biodiverse region. Northern regions are most water scarce, with just 1 % of renewable water resources but 18 % of the continental population, and water abstraction in the North already exceeds renewal rates. The Sahel, eastern, and southern regions are also water stressed, with 46 % of the continental population and 18 % of renewable water resources. These semi-arid regions are slated for a majority of the continent’s future development and also host distinct and world-renowned ecosystems. Many of the continent’s largest sustainability challenges are located here. Africa holds an estimated 0.66 million km^3^ of stored groundwater, much of which is non-renewable, fossil groundwater in large northern aquifers (MacDonald et al. 2012). The renewable groundwater resource is unknown and probably minimal in arid and semi-arid regions where water stress is highest. Consequently, the sustainable use of groundwater over most of the continent is likely confined to small-scale rural water supply and buffering against severe droughts (Edmunds 2012; MacDonald et al. 2012).

**Fig. 1:**
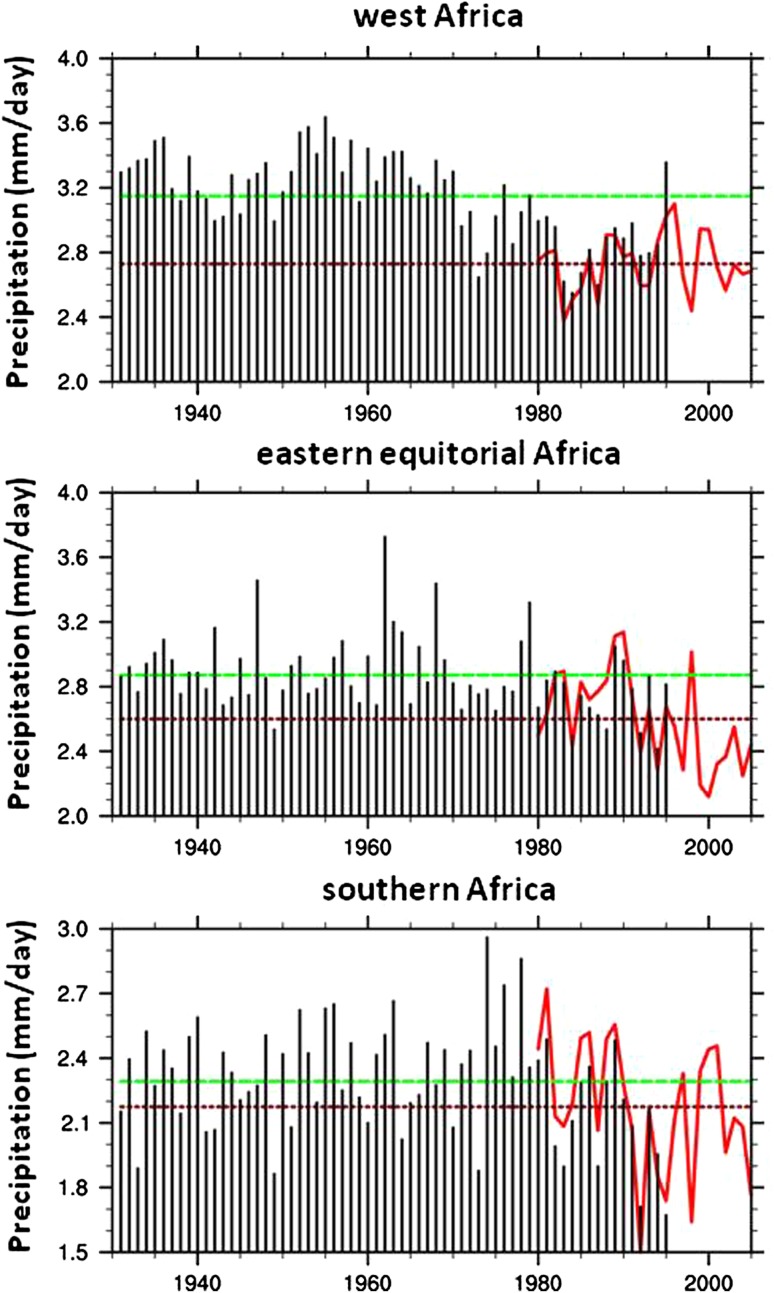
Regional averages of annual mean precipitation for three regions of Africa illustrating differential drying of the continent during the latter decades of the 20th century. *Black bars* are based on 1930–1995 data from the Climatic Research Unit of the University of East Anglia (Hulme 1992). More recent annual mean data (1979–2005) depicted by the *solid red line* come from the Global Precipitation Climatology Project (Huffman et al. 1997). The separation between the *solid green* and *dashed red horizontal lines* depicts the shift in annual means during the two time periods considered. From Giannini et al. (2008); used with permission

Regional limitations in renewable water resources are exacerbated by high inter-annual variability in precipitation. Precipitation in eastern and southern African is most variable, but over the last decades of the twentieth century western Africa showed extraordinary variability, punctuated by the western and central Sahel droughts of the 1960s and 1970s (Dai et al. 2004; Elagib and Elhag 2011). These droughts highlight what was recently shown to be a continental-scale process of drying (Giannini et al. 2008; Conway et al. 2009; Conway 2011) (Fig. 1), but years of anomalously high precipitation also occur in the rainfall record in eastern and southern Africa (Conway et al. 2005). Recent global monitoring and improved modeling techniques reveal that the combined influences of sea surface temperature in the Atlantic and Indian Oceans, modulated by ENSO, control the observed trends. Warming of the tropical oceans and cooling of the North Atlantic Basin are correlated with continental-scale drying, while warm ENSO events more strongly influence drying in eastern and southern regions by stabilizing the atmospheric column and reducing convection and associated precipitation (Giannini et al. 2008; Ambrosino et al. 2011). An exception to this pattern occurs in years of positive-phase circulation dipole events in the Indian Ocean, when unusually warm sea surface temperatures off the eastern coast of Africa are correlated with heavy precipitation events, normally during the so-called short rains falling from October to December (Conway et al. 2005). This phenomenon is thought to explain the unprecedented rains of 1961–1962, which caused widespread flooding in eastern Africa and a sudden 2 m rise in the level of Lake Victoria (Piper et al. 1986).

**Fig. 2:**
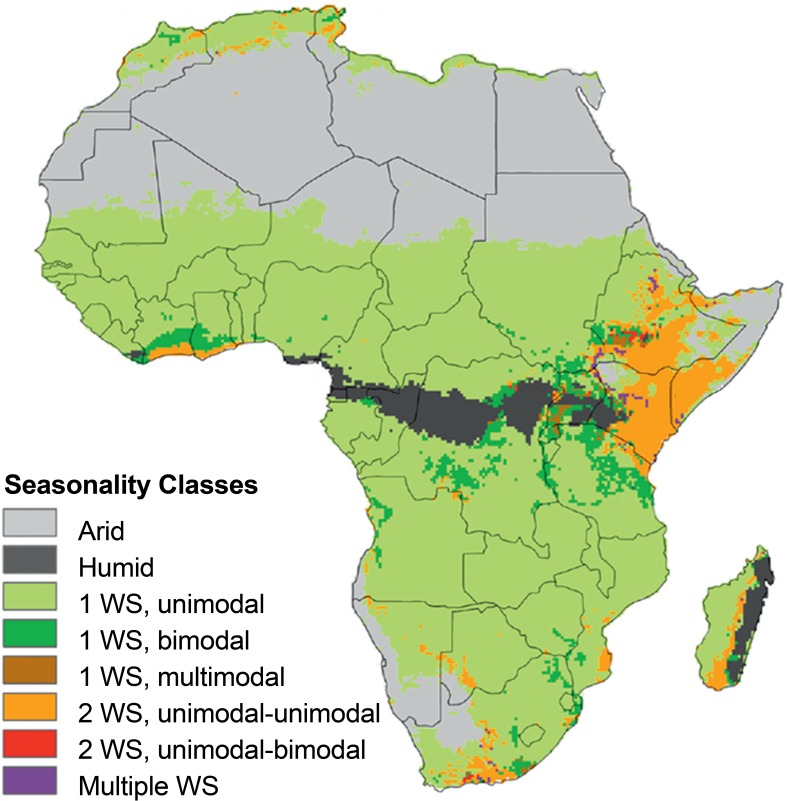
Rainfall seasonality patterns based on monthly accumulated rainfall estimates from the Tropical Rainfall Measuring Mission Multisatellite Precipitation Analysis (TMPA) for 1998–2010 (Huffman et al. 2007). The predominant patterns are a single wet season (1 WS unimodal or bimodal) north and south of the humid tropical rain belt and a dual wet season (2 WS unimodal–unimodal) in eastern tropical Africa. Other more complex seasonality patterns also occur in isolated areas. From Herrmann and Mohr (2011); used with permission

More predictable, but still variable, seasonal rainfall patterns are related to oscillations in the inter-tropical convergence zone, which produces a unimodal, monsoon-type rainfall regime north and south of the equator and a bi-modal regime along the equator, especially in eastern Africa (Fig. 2) (Herrmann and Mohr 2011). Persistent spatial rainfall patterns are also strongly influenced by topographic features. Mountains and highlands act as water towers collecting excess rainfall and conveying water to downstream arid and semi-arid areas in rivers (Vörösmarty et al. 2005; Viviroli et al. 2007; UNEP 2010; Lopez-Moreno et al. 2011). The most regionally significant examples are the Ethiopian Highlands and East African Rift Zone feeding the Nile River, the Lesotho Highlands feeding the Orange and Limpopo Rivers, and the Atlas Mountains feeding coastal rivers of Morocco. The Niger and Zambezi Rivers are also important conveyors of excess rainfall from the tropical African rainbelt to downstream semi-arid areas to the northwest and southeast, respectively (Collins et al. 2011). Basin scale analyses of rainfall-runoff relationships reveal that inter-annual variability in river flows is larger than, but linked to, variability in precipitation. Annual river flows in semi-arid western, eastern, and southern Africa decreased over the last decades of the twentieth century in response to decreasing regional precipitation (Conway et al. 2009). No comparable analysis of groundwater dynamics is yet available at regional scales or over decadal time-scales, but local studies confirm relationships between decreased precipitation, falling water tables, and declines in river runoff (Taylor et al. 2009).

**Fig. 3:**
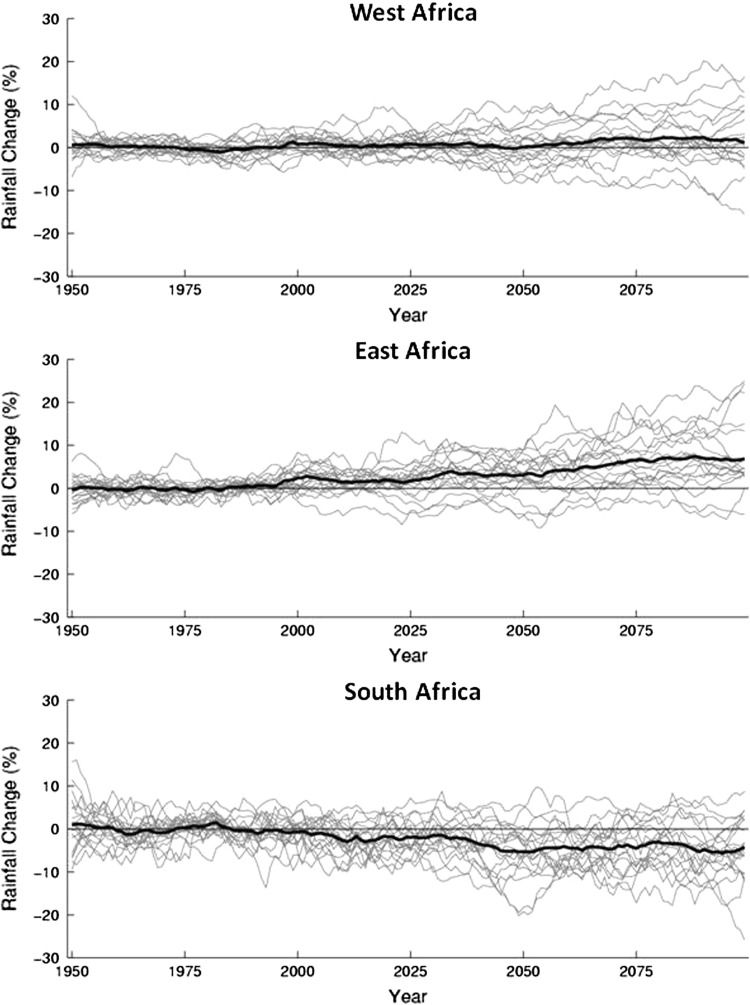
Regional averages of precipitation from 1950 to 2100 as simulated in IPCC 4AR. *Gray lines* depict results of individual models while the *heavy black line* depicts the multi-model mean. 2000–2100 simulations based on A1B scenario. From Giannini et al. (2008); used with permission

Given the constraints on availability of renewable water resources across many regions of Africa, future climate change must factor prominently in development planning. Recent multimodel ensemble simulations of temperature and precipitation trends over the next century provide an indication of what is to come, but projections of individual models vary due to remaining uncertainties in the relative importance of different controlling mechanisms mentioned above (Conway 2011). Average results reveal no clear future trend in annual precipitation for western Africa or the Sahel region, but precipitation in eastern and southern Africa is expected to increase and decrease, respectively (IPCC 2007; Giannini et al. 2008; Conway 2011) (Fig. 3). More regionally focused multimodel ensembling, which weighs model outputs using a Bayesian approach, is providing detailed projections of both mean and extreme changes in precipitation (Shongwe et al. 2009, 2011). Projections for southern Africa are disconcerting and indicate an 11 % reduction in austral summer (wet season) rains by 2100 and increased frequency of drought over western parts of the region, including the Kalahari Desert and adjacent areas (Shongwe et al. 2009). In eastern parts of southern Africa mean precipitation during peak summer months is not projected to change significantly, but rains are expected to begin later and end earlier. In fact, the summer rainy season is projected to become shorter across all of southern Africa. Winter (dry season) rains are also projected to decrease by 20–45 % across the entire region (Shongwe et al. 2009). By contrast, mean precipitation over equatorial eastern Africa is projected to increase by more than 10 % in most areas, with decreasing frequency of droughts and increasing frequency of extreme wet years (Shongwe et al. 2011). Future projections of river discharges track precipitation with increasing and decreasing flows in eastern and southern Africa, respectively, and no clear trend in western Africa or the Sahel (Conway et al. 2009). In semi-arid regions, reductions in rainfall may result in far higher percentile reductions in surface runoff. For example, in an area with an annual precipitation of 500 mm, a 10 % reduction in rainfall may lead to a 50 % decrease in runoff (de Wit and Stankiewicz 2006). Continued high seasonal and interannual variability in runoff is also expected.

High spatial and temporal variability of renewable water resources across Africa has been a challenge to human development for millennia and will remain a challenge in the future. Sustainable development of the continent’s water resources therefore requires understanding, adapting to, and working within the limits imposed by the continent’s climate, renewable water resources, and environmental water needs.

## Projections for Increased Water Use in Food and Energy Production

Projections for increased water use in Africa are dominated by agriculture and hydropower. Increased consumptive use by agriculture applies to both irrigated and rainfed crops. Increased non-consumptive use by hydropower is dominated by plans for medium and large projects; small hydropower is also touted, but reliable information on installed or potential capacity was not found during this review. Most irrigated agriculture and hydropower generation requires infrastructure to store water during wet periods for later use during dry periods or droughts. Thus, multipurpose dams factor prominently in development plans. Several recent studies have investigated the magnitudes of increased water use expected in the coming decades, as well as its spatial arrangement.

**Fig. 4:**
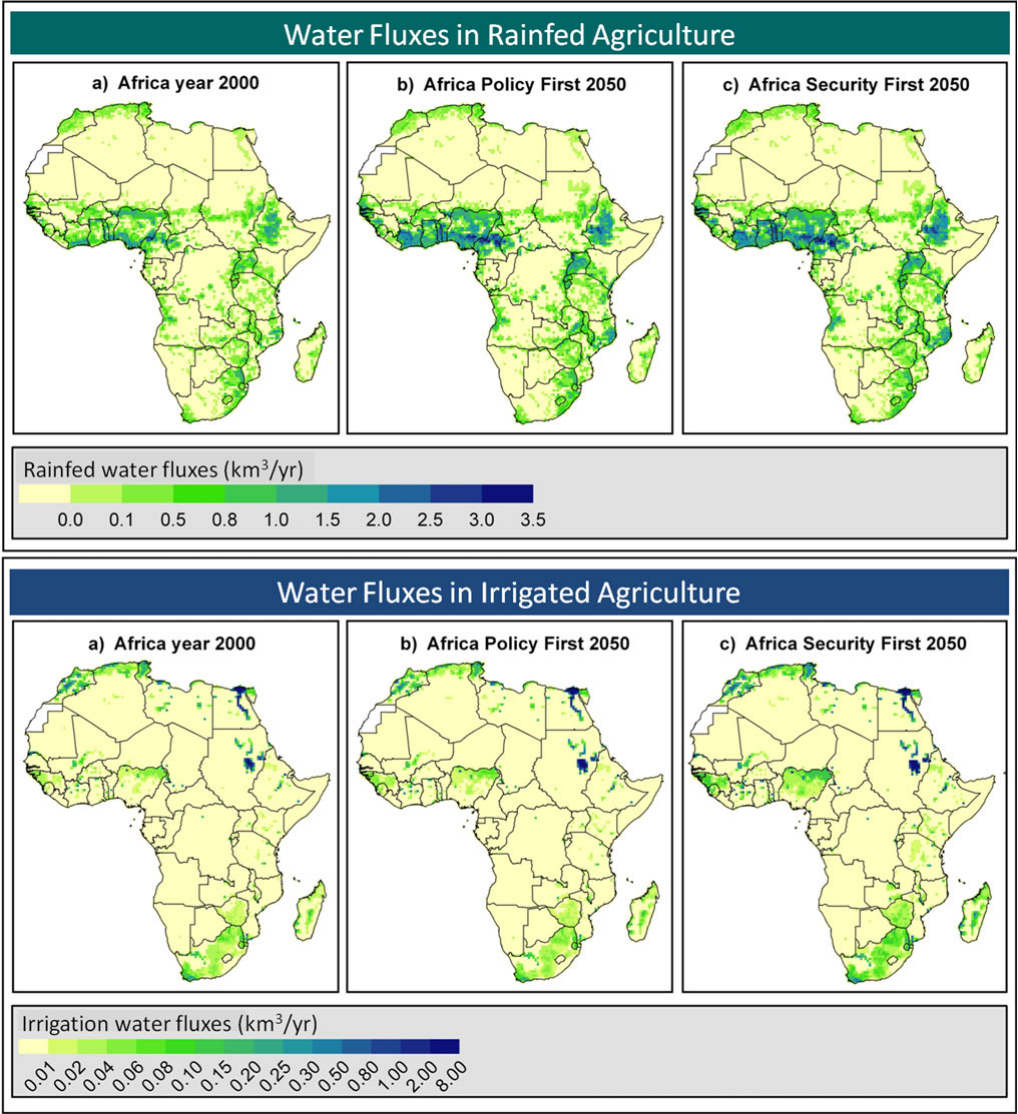
Simulated annual transpiration water fluxes related to rainfed (**a**) and irrigated (**b**) crops in the years 2000 and 2050, reflecting expected agricultural expansion and intensification in western, eastern, and southern regions. Simulations for 2050 are based on Policy First and Security First scenarios of the UNEP Global Environmental Outlook-4 Report (GEO-4) (Rothman et al. 2007). Policy First is a more optimistic scenario based on a high level of international cooperation in development and implementation of strong policies to protect the environment while pursuing economic growth. Security First is a more pessimistic scenario with less cooperation and more intense resource exploitation and fewer environmental protections. From Weiss et al. (2009); used with permission

Beginning with agriculture, the FAO estimates crop production must increase at 2 % per year to meet growing food requirements of sub-Saharan Africa over the next 40 years (Bruinsma 2009). About 75 % of growth is expected to come from intensification in the form of yield increases (69 %) and higher cropping intensities (6 %) and 25 % of growth is expected to come from expansion of agricultural lands. In total FAO expects agricultural land, estimated at 2.4 million km^2^ in 2009, to increase approximately 600 000 km^2^ by 2050 (Table 2). These FAO projections are conservative when compared with assessments of the Global Water Systems Project and CGIAR Challenge Program on Water and Food, which project expansion of agriculture by 1.0–1.4 million km^2^ over the same time period (Weiss et al. 2009; de Fraiture and Wichelns 2010) (Fig. 4). Projected increases in the area of irrigated agriculture range from 20 000 km^2^ (Bruinsma 2009) to 94 000 km^2^ (Weiss et al. 2009), with associated increases in water withdrawals of 19 and 82 %, respectively, over the estimated 2009 value of 185 km^3^/year (FAO-Aquastat 2009) (Table 2). Using revised FAO figures for irrigated area in Africa that consider informal as well as formal irrigation schemes, You et al. (2011) have recently estimated that irrigated agriculture could be profitably expanded by nearly 150 000 km^2^ over the next 50 years for a total of nearly 220 000 km^2^. Approximately 70 % of the total is expected come in the form of large-scale irrigation systems. The greatest potential for expansion is in Nigeria, Benin, Guinea, Mozambique, Sudan, Ethiopia, and Tanzania (You et al. 2011).

**Table 2:** Estimates of existing and projected extent of cropland and volume of irrigation water use in Africa

	FAO^a^	GWSP^b^	CGIAR^c^
Total cropland (km^2^)	2 200 000	2 292 000	1 580 000
Total irrigated cropland (km^2^)	72 000	235 428	60 000
Total cropland 2050 (km^2^)	2 800 000	2 543 000–3 808 000	1 690 000–2 520 000
Total irrigated cropland 2050 (km^2^)	92 000	308 847–329 606	70 000–130 000
Present water consumption by irrigation (km^3^/year)	105	179	64
2050 water consumption by irrigation (km^3^/year)	140	194–330	100–159

Difference between total and irrigated cropland reflects area of rainfed crops

^a^Bruinsma (2009)

^b^Weiss et al. (2009). Range of 2050 projections based on scenarios of the fourth Global Environment Outlook: Environment for Development (GEO-4) assessment (Rothman et al. 2007)

^c^de Fraiture and Wichelns (2010). Range of 2050 projections based on optimistic scenario assuming 80 % of yield gap is bridged and a pessimistic scenario assuming only 20 % is bridged. See cited references for details

While increased irrigation factors prominently in agricultural development in Africa, greater than 90 % of African agriculture is, and will remain, mostly rainfed. Estimates are that the water requirements of African rainfed crops total approximately 1100 km^3^/year (Weiss et al. 2009; de Fraiture and Wichelns 2010), with peak use in more humid zones of western, eastern, and southern Africa where abundant cropland occurs (Weiss et al. 2009; Liu and Yang 2010). Over the next 40 years, projections of the expansion of rainfed agriculture are as high as 1.4 million km^2^, with corresponding increases of soil water use of 955 km^3^/year (Weiss et al. 2009). The explicit addition of water transpired by rainfed crops (so-called green water) to calculations of managed agricultural water has focused increased attention on this resource and is positively influencing programs to enhance water productivity of rainfed crops (Rockström et al. 2009). While irrigated agriculture depends on water stored in reservoirs (large and small) and aquifers, rainfed agriculture depends on water stored in the soil column. Innovations in soil water capture and storage are likely to make the greatest cumulative contribution to sustainable agriculture in Africa. Additional attention is devoted to this topic later in the article.

Turning to future hydropower development, estimates are that between 75 and 500 GW of new power generation capacity are needed to provide universal electricity coverage in Africa, depending on the level of service provided, and the Africa Development Bank has set a target of 102 GW of new capacity by 2030 (ADB 2008). The contribution of hydropower to the total is uncertain, but estimates are that the economically viable hydropower potential of the continent is between 220 and 280 GW (Eberhard et al. 2011; Kumar et al. 2011). Hydropower accounts for 35–50 % of current capacity (Eberhard et al. 2011; WWAP 2012), a majority of which is concentrated in the DRC, Zambia, Nigeria, Ghana, and Cameroon. The largest existing hydroelectric facilities are Aswan (2100 MW) on the Nile, Cahora Bassa (2075 MW) and Kariba (1320 MW) on the Zambezi, and Akosombo (1020 MW) on the Volta. The largest single project under development is Grand Inga on the Congo River in the Democratic Republic of Congo (DRC), which is also the largest conceived project in the world at an estimated potential of more than 35 GW. Because hydropower potential is focused in a limited number of sites, efficient and potentially sustainable development of hydropower depends on cooperation and benefit sharing among countries. Power pools have thus been established in Central, East, West, and Southern Africa regions, which bring potential benefits in the form of reduced costs and environmental impacts but present many challenges for transboundary cooperation (Eberhard et al. 2011).

**Fig. 5:**
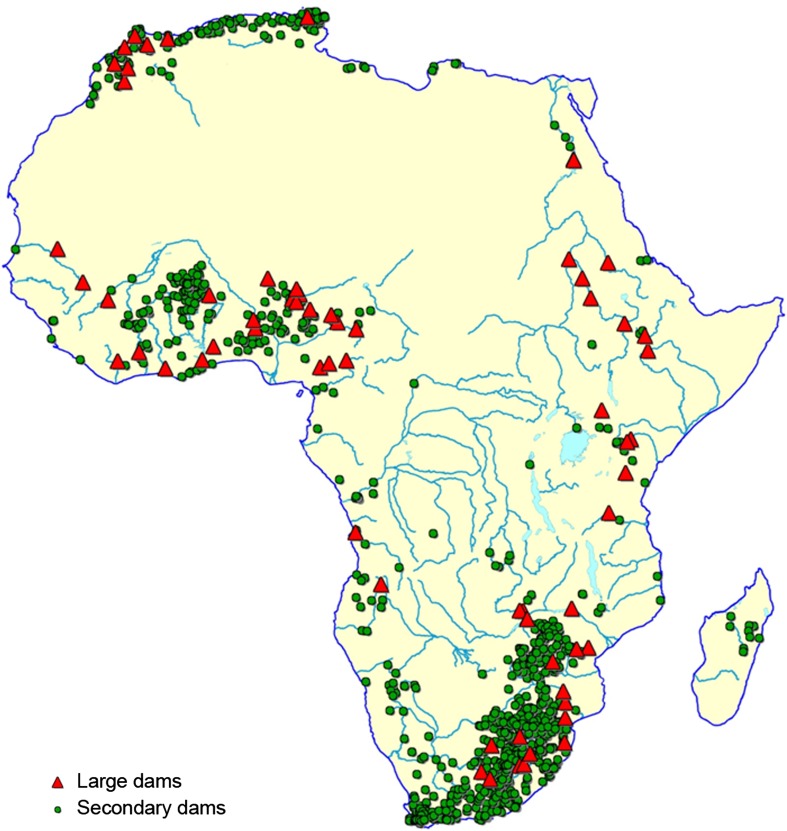
Location of 135 large dams (≥500 000 m^3^) and 1072 secondary dams (<500 000 m^3^) based on data from FAO-Aquastat. From Darwall et al. (2011); used with permission

Africa has a surprisingly large number of dams already, given the currently low levels of access to water supply, irrigation, and hydroelectricity. Recent high-resolution mapping of the world’s reservoirs and large dams reports 700 dams and reservoirs for Africa with a storage capacity of nearly 1000 km^3^ (Lehner et al. 2011), and FAO reports well over 1000 dams on the continent (Fig. 5). Most are located in southern and northern Africa, but large numbers also occur on western rivers draining to the Gulf of Guinea. Most dams are multipurpose. In a recent analysis of 972 dams across the continent, irrigation is cited as a major purpose in 65 % of dams, followed by water supply (38 %) and hydropower (13 %) (Strobl and Strobl 2011). New capacity is planned through the construction of new dams and rehabilitation of existing dams. Approximately 150 proposed dams are documented in international databases, mainly for central and eastern regions and the Gulf of Guinea (You et al. 2011). New dams are expected to increase storage by approximately 32 %, and rehabilitation of existing dams could expand storage even further (You et al. 2011).

The contours of future water development are thus clear and include more water use and additional storage capacity of several types. Large dams bring measurable benefits in the production of hydroelectricity and supply of irrigation waters (Eberhard et al. 2011; Strobl and Strobl 2011), but there has been considerable criticism of how these benefits are shared, the social and environmental costs, and whether better alternatives might be available (WCD 2000; Hathaway and Pottinger 2008). Dams currently alter the flow regimes of an estimated 75 000 km of African river channels, nearly 7 % of the total (Lehner et al. 2011). Large rivers (discharge > 1000 m^3^/s) are most impacted, with 43 % of their extent regulated, and the Nile, Volta, and Zambezi are the most highly regulated of the large rivers (Nilsson et al. 2005; Lehner et al. 2011). Conversion of natural landscapes and land acquisition in the process of agricultural expansion also raise serious social and environmental concerns (Deininger 2011; Ogutu et al. 2011; Woodhouse 2012). Minimizing the environmental impacts of development requires understanding better the status of ecosystems on the continent and the relationships between ecosystem health and the factors that will be impacted by future development, namely availability of natural landscapes, fragmentation of rivers, and spatiotemporal modifications in environmental water availability (surface flows and groundwater levels).

## Ecosystem Integrity and Threats Posed to Biodiversity and Ecosystem Services by Development

Africa’s ecosystems evolved under high climatic variability and are thus adapted to cope with natural limitations. Additional limitations imposed by human activities, however, reduce the number of ecological responses available to species, and the simultaneous pressure of multiple stressors weakens the resilience of whole ecosystems (Bouché et al. 2010; Hecky et al. 2010). Restricted movement is an example of one such human-induced limitation. For many large ungulates and associated predators in semi-arid regions, migrations are an effective adaptation to seasonal variations in water and food (Holdo et al. 2009; Cornélis et al. 2011). The migration of the wildebeest (*Connochaetus taurinus*) in the Mara-Serengeti ecoregion is one of the only enduring examples of what were once large migrations across many semi-arid regions (Harris et al. 2009), but there too the numbers of migrating organisms are in decline (Ogutu et al. 2011). Across the continent, wildlife has been confined to protected areas covering only a fraction of original ranges and recent indications are that conservation efforts are failing. Surveys indicate that large mammal population abundance declined by 59 % in protected areas between 1970 and 2005, due in large part to insufficient habitat and poaching (Craigie et al. 2010; Metzger et al. 2010; Fynn and Bonyongo 2011). Population losses were greatest in eastern and western Africa. Over approximately the same period, satellite monitoring reveals significant reductions in natural dry forests in southeastern Africa and savannas in eastern regions and the Sahel, driven by a 57 % increase in the area of agriculture (Brink and Eva 2009). Impinging development on areas of high conservation value, reduced flows of rivers passing through conservation areas, and further fragmentation by roads and fences are the most ubiquitous threats facing Africa’s natural landscapes and these pressures, if unchecked, will continue to grow through the twenty-first century (Holdo et al. 2011).

**Fig. 6:**
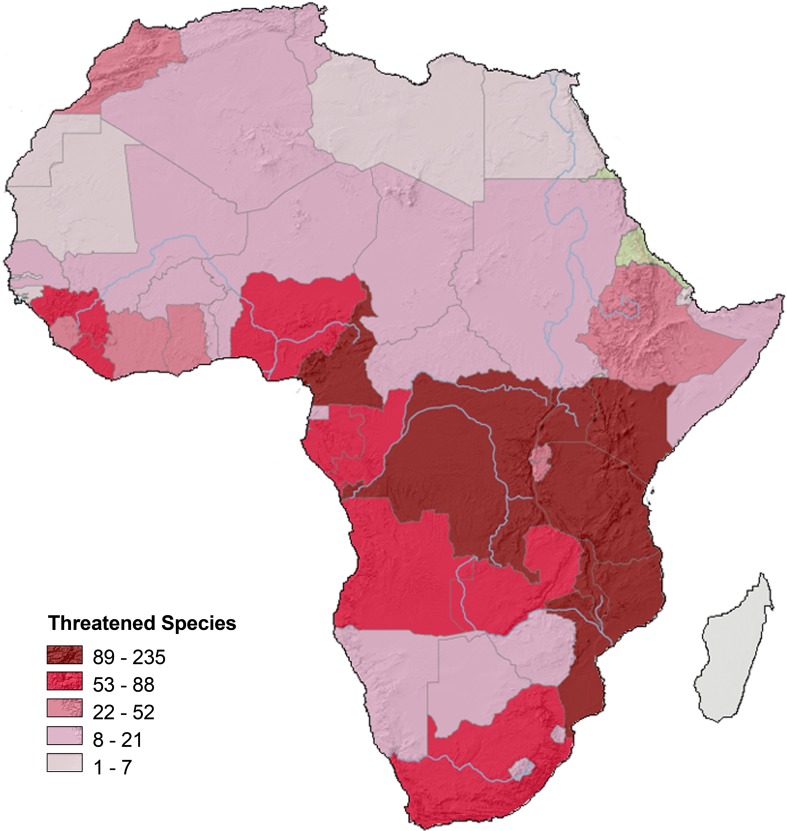
Richness of threatened species based on occurrence in countries, including fish, odonates, crabs, molluscs, and plants. Areas with the largest concentrations of threatened species include high-biodiversity equatorial regions and areas of western, eastern, and southern Africa targeted for increased dam building and water abstractions. From Darwall et al. (2011); used with permission

Africa’s aquatic ecosystems also exhibit high levels of natural resilience, including migratory habits and some remarkable adaptations of individual organisms to survive dry periods (Helfman 2007; Laleye and Entsua-Mensah 2009; Vijverberg et al. 2009). But human pressures on aquatic ecosystems are intense in Africa, just as they are in the rest of the world (Dudgeon 2010; Strayer and Dudgeon 2010). The continental richness of Africa’s freshwater diversity and the mounting threats are detailed in the results of a 6-year assessment by the IUCN Species Program (Darwall et al. 2011). The survey confirmed, and highlighted, high levels of aquatic biodiversity in Rift Valley Great Lakes (Lake Malawi, Lake Tanganika, Lake Victoria) and rivers of the Congo, with hundreds of mostly endemic species (Thieme et al. 2010; Snoeks et al. 2011). Species diversity in rivers of semi-arid savannas is much lower, commonly less than 50 species per river basin, but endemism remains high. The level of threat to aquatic species was greatest in developed semi-arid areas where pressures are already high on water resources, leading to insufficient flows in rivers, fragmented channels, riparian degradation, and pollution runoff (Holland et al. 2011) (Fig. 6), and in the Great Lakes, where the principle treats are pollution from catchment runoff, over-fishing, and introduction of exotic species (Hecky et al. 2010; Nyenje et al. 2010; Holland et al. 2011). Many wetlands fringing lakes and in river valleys have also been heavily impacted by human interventions, especially agriculture (Rebelo et al. 2010). Projections of the impacts of climate change and continued human pressures over the next 40 years indicate that changing hydrologic conditions will ultimately affect 80 % of African fish species (Thieme et al. 2010).

The case of the Lake Victoria Basin provides a stark example of these impacts. During the past 70 years, the human population of the 180 000 km^2^ lake catchment has grown from approximately 3.5 million to more than 30 million people, and pressures on land and water resources have grown proportionally (reviewed by Masese and McClain 2012). Water quality degradation in the lake and loss of biodiversity have received the most attention (Hecky et al. 2010), but unmanaged expansion of croplands, cattle grazing, urbanization, and infrastructure have also severely degraded the basin’s natural landscapes and rivers (Odada et al. 2009). Large wildlife (e.g., wildebeest, giraffe, and elephants) have all but disappeared from inhabited areas, and even in and around protected areas like Masai-Mara National Reserve in Kenya, large wildlife abundances have decreased by 50 % over the past 30 years (Ogutu et al. 2011). Increased erosion and nutrient runoff have contaminated rivers, and natural flow regimes and channel connectivity have been altered by dams, uncontrolled abstractions, and land use change (Masese and McClain 2012). Impacts on river and wetland ecosystems include biodiversity loss and alterations to fundamental ecosystem properties (Masese et al. 2009; Raini 2009). The potential consequences of these property shifts are not yet clear, but the combined effects of contamination and biodiversity loss have certainly reduced ecosystem services available to inhabitants of the basin.

The environmental impacts of much of the development that has taken place to-date are of concern because ecosystem services provided by Africa’s natural landscapes, free-flowing rivers, associated wetlands, and lakes are fundamental to the basic wellbeing of a large proportion of the population (MEA 2005; Holland et al. 2011). Given the level of development across most of the continent, African’s rely heavily on the ecosystem services to regulate soil fertility beneath agricultural fields and grazing lands and even to provision food gathered from adjacent natural areas. Biomass currently accounts for more than 80 % of energy consumption in Sub-Saharan Africa (excluding South Africa) (WWAP 2012). Freshwater ecosystem services are especially valued, first for the provision of domestic and agricultural water. People also harvest 45 % of all know African fish species, mainly for human consumption, and 58 % of aquatic plant species, mainly for a variety of non-food uses (Sieben 2009; Ghogue 2011; Holland et al. 2011). Mollusks and crabs are locally important (Holland et al. 2011). Less visible services contributing to wellbeing include groundwater recharge, assimilation of contaminants (especially nutrients and organic wastes), and storage of carbon. It is essential, therefore, that ecosystem services be preserved as development progresses.

## The Way Forward—Promising Research to Inform Decision Making and Aid Implementation

The direct dependency of Africans on ecosystem services remains high, and tension between necessary exploitation and pragmatic conservation will challenge environmental sustainability for decades to come. Fundamental principles of sustainable development and conservation of biodiversity are well represented in international and national laws, and these principles are familiar to decision makers at many levels. The nature and magnitude of the problems have also been delineated by several recent large-scale science initiatives (Abell et al. 2008; Rockström et al. 2010; Vörösmarty et al. 2010; Darwall et al. 2011). Thus, the objectives are clear. Agricultural productivity must increase in a manner that minimizes losses of forests and savanna ecosystems and reduces pressures on conservation areas, and abstractive (irrigation) and in-stream (hydropower) water use must increase in a manner that minimizes degradation of aquatic ecosystems. Continued efforts at good policy-making, awareness raising, and information gathering are important, but a pressing need for resource managers is scientific and socioeconomic guidance in concrete and appropriate actions to realize these goals and capacity building to enable the implementation of these actions. Practitioners generally understand “what” needs to be done but continue to ask “how” to do it? The following paragraphs highlight areas of current applied research that hold special promise in the pursuit of appropriate answers to the most pressing “how” questions.

Innovations in irrigation and agricultural water productivity are a dynamic area of research and the basis for a productive debate about the best approaches to be applied in Africa (Lankford 2009; van der Zaag 2010). A universal question in African agriculture, however, and one that is especially pertinent to the greater than 90 % of agriculture that is rainfed, is how to retain more rain water in the soil in semi-arid areas where water limits crop yields. Answers to this question can boost productivity for food security, build resilience to short-term drought, and minimize the required areal expansion of agriculture. The most optimistic scenarios project a doubling or tripling of cereal yields per hectare from improvements in rainfed agriculture (Rockström et al. 2009, de Fraiture and Wichelns 2010). Increased infiltration and reduced evaporative water losses (e.g., by mulching) are key to increasing soil water storage. In more arid regions, even greater improvements in yield are possible if rainfall is augmented by supplemental water harvested during rain events and stored in small reservoirs for use during dry periods (Wisser et al. 2010) or routed to fields during runoff spates and stored as soil moisture (Mehari et al. 2011).

Initial research into the benefits of small-system innovations in a dry portion of northeast Tanzania (precipitation of approximately 600 mm/year) indicates yields of maize are improved by increasing the water storage capacity of the soil through deep tillage and the application of mulch to reduce evaporation, although increases were modest (max 17 %) or even reduced if mulch was not applied to control evaporation (Enfors et al. 2011). Much higher yields were obtained (up to 400 % of control) when rainwater was harvested and directed onto terraced fields (Makurira et al. 2011). Similar increases in yield have been recorded among different crops in larger spate irrigation systems in arid zones of Ethiopia (Steenbergen et al. 2011). Improved nutrient management (especially nitrogen) and selection of proper crop varieties are also essential to improving productivity of rainfed agriculture (van der Zaag 2010). Decisions about developing centralized large-scale storage or more distributed, farm-scale storage as described here depend on biophysical, economic, and social factors, but appropriate small-scale interventions are likely to have reduced environmental impacts (van der Zaag and Gupta 2008).

Increasing productivity of rainfed agriculture can make an important contribution to meeting future food needs and economic development while minimizing demands on surface water and groundwater sources, but it must be done within a well implemented framework of adaptive land management that reduces impacts on biodiversity and even supports the ecological functioning of protected areas. Otherwise increased productivity will likely promote greater expansion for economic gain rather than reducing pressure on natural lands (Ewers et al. 2009). This is especially important in light of the recent acceleration of land acquisition for agriculture (Deininger 2011; Woodhouse 2012). In many parts of Africa, crops are interspersed with grazing lands, game reserves, woodlands, and forests. In the least developed regions, boundaries are often poorly defined, with humans benefiting from surrounding ecosystem services and wildlife moving throughout the landscape matrix. These agricultural landscapes are prime candidates for the implementation of ecoagricultural approaches to improve agricultural productivity while at the same time mimicking and supporting the ecological structure and function of interspersed natural lands (Scherr and McNeely 2008; Brussaard et al. 2010). This approach is appropriate for establishing buffer areas around, and corridors between, protected areas. There are even encouraging recent data to suggest that migrations of large ungulates can be restored if acceptable corridors are established between required habitat types (Bartlam-Brooks et al. 2011).

Conservation of aquatic ecosystems over the coming decades is especially challenging due to the nearly ubiquitous threats these systems face (Dudgeon 2010; Dudgeon et al. 2011) and the fact that protected areas are poorly suited for the conservation of aquatic ecosystems (Linke et al. 2011; Nel et al. 2011a). Construction of new dams (large or small) will impact river connectivity and alter flow regimes across the continent and increased water abstractions may drain rivers and wetlands in the Sahel and eastern and southern semi-arid regions. Averting the potentially devastating ecological consequences of these interventions requires determining and implementing environmental flow allocations. Environmental flows describe the quantity, timing and quality of water flows required to sustain freshwater ecosystems at desired levels of ecological functionality. In water policies of southern and eastern Africa, environmental flows are combined with minimum flows for basic human needs, constituting a “reserve flow” that is of highest priority in allocation planning (South Africa National Water Act 1998; Kenya Water Act 2002; Tanzania Water Resources Management Act 2009). Considerable progress was made in the assessment of rivers across South Africa during the past two decades, and more recent assessments have been completed in Ethiopia, Kenya, and Tanzania (Kashaigili et al. 2007; McCartney et al. 2009; PBWO/IUCN 2009; LVBC and WWF-ESARPO 2010). Environmental flow regimes preserve key components of a river or wetland’s annual flow variability, including necessary baseflows in different seasons, important floods that trigger ecological responses or shape channel morphology, and even annual droughts that limit invasions of harmful exotic species (Arthington et al. 2010).

To facilitate the widespread application of environmental flow assessments, a collaborative framework has been recently developed to simultaneously assess the environmental flow needs of rivers across large areas (Poff et al. 2010), and environmental flows are being integrated into freshwater conservation planning (Nel et al. 2011b). In highly regulated rivers with one or more significant dams, environmental flow requirements can be met by releasing water from reservoirs in accordance with flow recommendations and setting back levees to enable as much floodplain inundation as possible (Richter and Thomas 2007). Re-operating dams used for hydropower is more complex because releases are already timed to meet electricity demands and changes may have significant economic consequences (Tilmant et al. 2010). Ideally consideration of environmental flow requirements will be built into the initial design of dams and other control structures (Higgins et al. 2011), but a range of post-construction measures have also been proposed, such as construction of smaller re-regulation reservoirs downstream or pumped storage reservoirs upstream, each of which enables environmental flow releases into the river (Richter and Thomas 2007). Where environmental flows have been assessed, the bottleneck now is implementation, which is lagging in many countries due to lack of will, resources, and capacity (Le Quesne et al. 2010).

## Final Remarks—Governance and Financial Factors

The ambitions expressed in Africa Water Vision 2025 strike a prudent balance between development and conservation. Priority is first given to allocation of water for environmental sustainability (environmental flows), which protects an array of ecosystem services benefiting Africans today and into the future. Surplus water is prioritized for other productive uses, especially increased agricultural production and hydropower generation, both of which are under-developed in many regions. The abundance of surplus water, however, varies greatly in space and time across the continent and may often be at odds with current and future demand. This poses difficult challenges for the environmental sustainability of African development. This review examined a wealth of recent scientific results that delineate the scope and dimensions of these challenges. Moreover, it presented a growing body of knowledge and set of tools available to inform decision making and aid in implementation. The message is encouraging, and while there is much still to be learned, lack of knowledge, appropriate technology, and guidance tools should not be seen as a limiting factor in sustainable African development.

Many readers will note, however, the overwhelming importance of proper governance and economic growth/investment to achieve development goals in Africa. While outside the scope of this review, improvements to governance and strengthening of the financial base for water resources management are prominent themes in Africa Water Vision 2025 and were reaffirmed by the international community at the Rio +20 UN Conference on Sustainable Development (UN 2012). As with the scientific and technical aspects of water resources development in Africa, much research attention has recently been devoted to governance and financial aspects (Jacobs and Nienaber 2011; Schreiner and Hassan 2011; Hanson et al. 2012). Critical success factors include openness and transparency in decision-making processes and cooperation among countries. Cooperation is essential because virtually all of the continent’s water resources lie in transboundary basins and aquifers (UNEP 2010) and energy needs will be best achieved though regional sharing of hydropower (Eberhard et al. 2011). National and international sources are targeted for needed financing, including water users and private sector companies through the implementation of proper pricing and cost recovery mechanisms. There should be no illusions, however, about the magnitude of the challenges ahead and the need to effectively integrate scientific, technical, financial, and governance approaches.
